# Praxis Model for Technology Development: a participatory approach^*^

**DOI:** 10.1590/1980-220X-REEUSP-2023-0041en

**Published:** 2023-06-09

**Authors:** Cléton Salbego, Elisabeta Albertina Nietsche

**Affiliations:** 1 Universidade Federal de Santa Maria, Santa Maria, RS, Brazil.

**Keywords:** Nursing Research, Methods, Technological Development, Validation Study, Nursing, Investigación en Enfermería, Métodos, Desarrollo Tecnológico, Estudio de Validación, Enfermería, Pesquisa em Enfermagem, Métodos, Desenvolvimento Tecnológico, Estudo de Validação, Enfermagem

## Abstract

**Objective:**

To present the Praxis model for Technology Development validated content and appearance.

**Method:**

A methodological study with validity of a nursing research model, carried out from March to September 2022. A total of 26 research nurses from all regions of Brazil participated. The model items were considered relevant and reliable when the Content Validity Index Confidence Interval was ≥ 0.8 in just one round. When minor, modifications or deletions were made, as suggested by specialists.

**Results:**

The model was operationalized in the pragmatic, productive/artistic, experimental and revolutionary phases. Its assessment was considered relevant by judges, obtaining an average index of 0.950 for its content and 0.825 for appearance.

**Conclusion:**

The praxis model presents theoretical clarity, a relevant and applicable approach in technological development research in nursing.

## RESUMO

**Objetivo:**

Apresentar o conteúdo e aparência validados do Modelo Práxico para Desenvolvimento de Tecnologias.

**Método:**

Estudo metodológico, com validação de um modelo de pesquisa em enfermagem, realizado de março a setembro de 2022. Participaram 26 enfermeiros pesquisadores de todas as regiões do Brasil. Os itens do modelo foram considerados relevantes e confiáveis quando o Intervalo de Confiança do Índice de Validade de Conteúdo foi ≥ 0,8 em apenas uma rodada. Quando menor, modificações ou exclusões foram realizadas, conforme sugestões dos especialistas.

**Resultados:**

O modelo foi operacionalizado nas fases pragmática, produtiva/artística, experimental e revolucionária. Sua avaliação foi considerada pertinente pelos juízes, obtendo índice médio de 0,950 para seu conteúdo e 0,825 para aparência.

**Conclusão:**

O modelo práxico apresenta clareza teórica, abordagem pertinente e aplicável em pesquisas de desenvolvimento tecnológico em enfermagem.

## RESUMEN

**Objetivo:**

Presentar el contenido y apariencia validada del Modelo Praxis para el Desarrollo Tecnológico.

**Método:**

Estudio metodológico, con validación de un modelo de investigación de enfermería, realizado de marzo a septiembre de 2022. Participaron 26 enfermeros investigadores de todas las regiones de Brasil. Los ítems del modelo se consideraron relevantes y fiables cuando el Intervalo de Confianza del Índice de Validez de Contenido fue ≥ 0,8 en una sola ronda. Cuando fue menor, se hicieron modificaciones o supresiones, según lo sugerido por especialistas. Cuando fue menor, se hicieron modificaciones o supresiones, según lo sugerido por especialistas.

**Resultados:**

El modelo fue operacionalizado en las fases pragmática, productiva/artística, experimental y revolucionaria. Su valoración fue considerada relevante por los jueces, obteniendo un índice medio de 0,950 por su contenido y 0,825 por su apariencia.

**Conclusión:**

El modelo de praxis presenta claridad teórica, un enfoque relevante y aplicable en la investigación de desarrollo tecnológico en enfermería.

## INTRODUCTION

The Praxis Model for Technology Development (PMTD) has a theoretical/conceptual, visual and operational systematization to support the exploration, description, analysis, explanation, simulation and dissemination of different phenomena emerging from human activity in the multiple social scenarios of action of nursing. With a view to technological development (construction, validity and assessment), PMTD presents a pragmatic structure that allows the understanding of realities to assist in individual and/or collective praxis transformation. It is a methodological, scientific model applied to the resolution of problems arising from nurses’ practical activity, based on real needs, to achieve ideal results based on practical awareness and/or praxis.

Proposing technologies designed and applied in a practical context, with the active participation of the social actors for which they are intended, becomes an activity to be discussed by nursing in this research area. In a documental study, carried out in the Coordination for the Improvement of Higher Education Personnel (CAPES – *Coordenação de Aperfeiçoamento de Pessoal de Nível Superior*) Bank of Dissertations and Theses, it showed that until 2020 1.,733 researches with technological scope were published; however, 73 presented participative methodological potential, with greater highlight for studies of the last decade. With this, it was observed that the technologies produced until a certain moment did not demonstrate participatory bases.

Starting from this theoretical immersion, associated with the theoretical-epistemological framework^(1)^, PMTD was built, aiming to organize and indicate the conceptual and methodological structure to allow researchers to recognize the real needs of the population studied and, thus, develop an ideal product for the given problem.

It is necessary to (re)think technological production so that it is planned and applied in/for the practice of the audience for which it is intended. This movement allows thinking about the humanist, conscious and ethical character involved in the creation process^(1,2,3)^. In this context of praxis, onto-epistemological aspects^(4)^ are involved in the construction of knowledge. It becomes necessary for individuals to analyze/reflect/intervene in order to align problems of practical and human reality (ontological) with the knowledge of subjects and their sources and their ways of producing scientific knowledge (epistemological)^(4)^.

In the literature, several models of research are found that help scientific evolution in different areas. In nursing, for technological development, promising methodological possibilities are evident to respond to researchers’ needs^(5–7)^. However, the challenge lies in choosing models with participatory approaches, which encourage dialogue between subjects.

In this context, the development of technologies needs to start from methodological references that help researchers in the collection, interpretation and analysis of phenomena, contributing to the quality of productions. This initiative contributes to consistency between methodology, research problem and field of investigation^(8)^. Therefore, the present study, by presenting PMTD, will help researchers in choosing a participatory methodological reference option for nursing research.

Participatory methodologies stand out as promising, allowing research to respond to the community’s needs, in order to provide an active role for participants, with a view to developing emancipatory skills for transforming realities^(8)^. Based on these assumptions, this paper aims to present the PMTD validated content and appearance.

## METHODS

### Study Design

This methodological study aimed at validating the content and appearance^(9)^ of a research model for the development of nursing technologies.

### Location and Selection Criteria

The survey was carried out virtually, nationwide in the five regions of Brazil (North, Northeast, Midwest, Southeast and South).

Expert judges were intentionally selected according to their experience and proven knowledge in theoretical, epistemological and technological development in nursing, using Fehring’s criteria adapted to research interests^(10,11)^. The search was carried out by consulting the curricula available on the CAPES *Lattes* Platform, publications in journals or by indication of experts.

### Population, Sample and Data Collection

A total of 189 researchers from all regions of Brazil were invited to participate in the survey, through contact by email or WhatsApp^®^. They received a link to access Google Forms^®^, containing the Informed Consent Form, answered anonymously. Acceptance was obtained from 52 expert judges.

Subsequently, a link was sent to access the validity instrument hosted on Google Forms^®^. To validate the PMTD content and appearance, PMTD’s degree of relevance^(12)^ and reliability^(13)^ were analyzed based on the 30 questions, scored on a Likert-type scale with five levels of judgment: (1) strongly disagree; (2) partially agree; (3) neither agree nor disagree; (4) agree; and (5) strongly agree^(14)^. Finally, the sample of this study comprised 26 researchers who answered the research instrument within the established period. Data collection took place from March to September 2022. The judges proceeded with validity considering the model’s pertinence and reliability regarding its concepts, theoretical and philosophical basis, epistemological structure, operability of its phases and reproducibility.

### Data Analysis and Processing

To validate PMTD, the Content Validity Index (CVI) was used, which assesses the proportion or percentage of judges in agreement on certain aspects of the instrument and its items. The Level Content Validity Index (I-CVI) was applied to assess the agreement among judges in each assessment item, being calculated from the number of judges assessing the item as relevant and very relevant. The Scale-Level Content Validity Index, Average Calculation Method (S-CVI/AVE) was assigned to measure the proportion of scale items assessed as relevant and very relevant for each judge. Items with CVI ≥ 0.80 were validated^(12)^. To analyze whether the proportion of agreement regarding PMTD adequacy and relevance was statistically equal to or greater than 0.8, the binomial test was performed with a significance level of 5%^(15)^, i.e., a 95% Confidence Interval (95%CI). To verify the instrument’s internal consistency, Cronbach’s alpha coefficient was calculated^(13)^.

### Ethical Aspects

The present study was carried out in accordance with the norms of Resolutions 466/2012 and 674/2022 and Circular Letter 2/2021, belonging to the Brazilian National Health Council, approved by the Research Ethics Committee of the participating institution, under Opinion 4.856.484/2021. Participants were informed about the objectives of the study and, after expressing interest, they signed the Informed Consent Form (ICF).

## RESULTS

### Basic Concepts Applied to the Praxis Model

**Figure 1.**
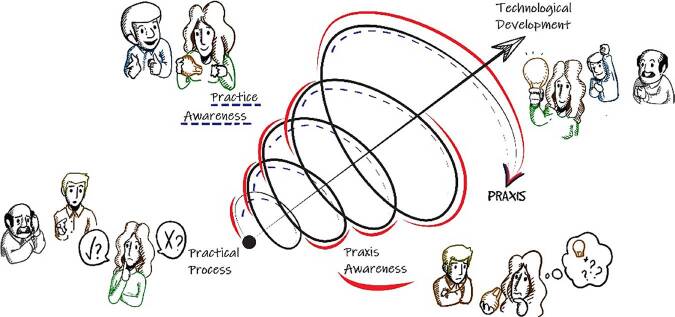
Representative spiral to technological development in the light of praxis – Santa Maria, RS, Brazil, 2023.

With a view to technological development in the health area, especially in nursing, PMTD presents a pragmatic structure that aims to guide the understanding of realities to help transform individual and/or collective praxis. The model has as its theoretical-philosophical basis human praxis and its awareness, whether practical or praxis (Figure 1).

Praxis is every conscious action that generates transformation. Awareness is the ability of human beings to accumulate knowledge (in the broad sense of term, values, habits, cultures, among others). It is not innate, it is potency, as it develops in the relationship between men and between them and the environment (Figure 1).

The practical process is characterized as a set of acts, (inter)relationships between active subjects (agents) and between them and the environment in which they are inserted. The practical process comprises the activity (human beings’ action) in the face of phenomena emerging from its context. It characterizes the agent’s starting point in/for the effective generation of solutions (technological development) (Figure 1).

Practical awareness is inserted as the ideal activity desired by man, which materializes allowing the transcendence of awareness in order to boost the creative act, i.e., awareness materialized in technological development (Figure 1).

Praxis awareness can contribute to enrich real material activity. In this tension between the ideal and the real, we can perceive the elevation of practical awareness to praxis, as the phenomenon called practical self-awareness will occur^(1)^.

Practical awareness and praxis awareness cannot be treated as similar, as they play different roles, but converge at a given moment in the practical process. This awareness is not separated, it is just at different levels of action in the practical process.

Researchers and researched collectively involved in the same practical process, awakening levels of practical awareness or individual and collective praxis awareness, will be able to operate together the technological development of a given ideal product (Figure 1).

### Phases for Implementing the Practical Technology Development Model

**Figure 2.**
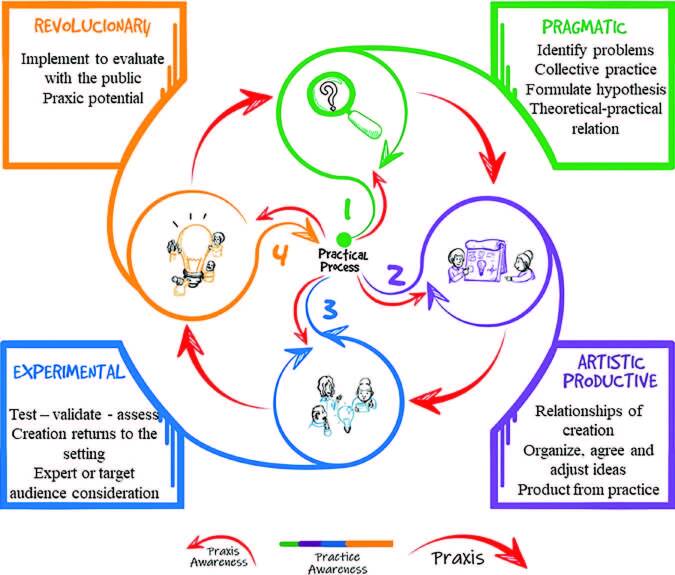
Representation of the Praxis Model for Technology Development phases – Santa Maria, RS, Brazil, 2023.

Sustained by the practical awareness of developing technologies by Brazilian nurses and allied to the praxis awareness subsidized by Adolfo Sanchez Vázquez’s framework on human praxis, PMTD is operationalized in four phases: pragmatic, productive/artistic, experimental and revolutionary (Figure 2). These phases are complementary and interrelated, allowing the revision of hypotheses at any time.

### Pragmatic Phase

This phase symbolizes the insertion of the researcher in the practical field, aiming at observation/reflection, understanding/interpretation of lived reality, knowing the social actors involved, their knowledge and practices, questioning themselves and the context in planning solutions.

**Figure 3.**
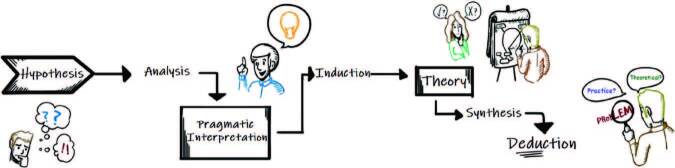
Representation of the path for conducting the pragmatic phase – Santa Maria, RS, Brazil, 2023.

The pragmatic phase represents a path to be followed for insertion in the practical universe and synthesis of the knowledge acquired. This will subsidize developing technologies, which usually takes place through a sequential order: (1) deduction; (2) analysis; (3) induction; and (4) synthesis (Figure 3). They permeate the path to be followed in this phase, characterized by the elaboration of research hypotheses, followed by the pragmatic interpretation of the investigative scenario and, finally, the pragmatic theorization which will result in the preliminary synthesis of the practical process’ emerging needs.

For the elaboration of hypotheses, it becomes possible for the researcher to construct affirmations about observed reality. It is necessary to clearly specify the problem to be investigated, be it theoretical or practical. At this stage, hypotheses aim to provide the connection between theory and practice, fact and investigation. It is characterized as a challenge, as it is necessary to define the basic concepts of the problem, which will be the lens for pragmatic observation.

In the pragmatic interpretation, purposes are created. We can say that these purposes are the justification or real need of the scenario for creating specific practical possibilities. These can constantly reveal themselves, permeating pragmatism, reaching the revolution of the universe, i.e., when new solutions are applied to the scenario of interest. Designing purposes for creation means involving high levels of practical awareness. Creating based on purposes requires reflexivity, characterizing praxis awareness.

Theorizing is complementary to previous phases and is believed to be guided by pragmatism. At this juncture, practice and theory must be intertwined, with a view to grounding thinking, reflecting, criticizing and acting in the face of the practical process. Theorizing is when theoretical-scientific support is needed as a mediation strategy between empirical knowledge and the practical process. It is the moment to analyze the problem and question about what was observed.

### Raising Awareness Levels: Productive/Artistic Phase

This phase involves high levels of awareness (practical and/or praxis) in order to produce a potentially transformative solution for the practical process. From the synthesis carried out in the previous moment, there are subsidies to structure a product or technological process with pragmatic meaning.

**Chart 1.**
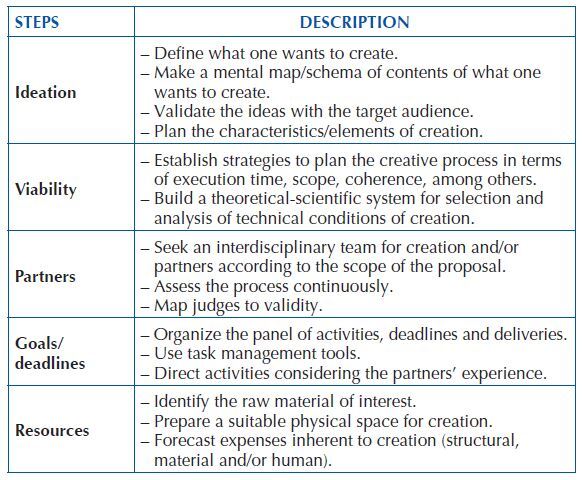
Structure of technological artistic production relations – Santa Maria, RS, Brazil, 2023.

Production relations are established with the aim of designing technological artistic production. They must follow an operational systematic, allowing to obtain control of the operations involved in the creative process, organized in ideation, feasibility, partners, goals/deadlines and resources (Chart 1).

### From Creation to Testing: Experimental Phase

**Chart 2.**
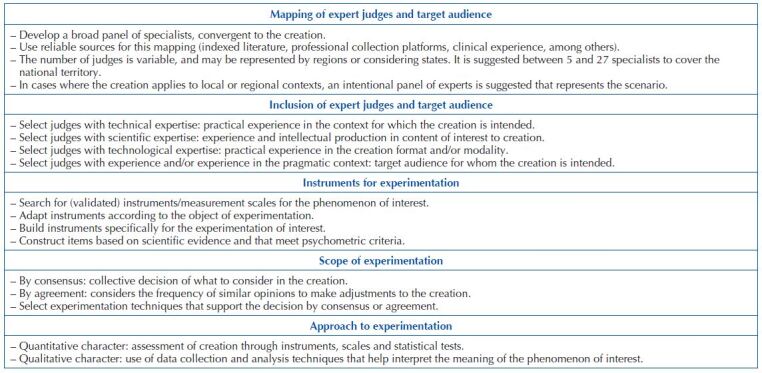
Script to organize the experimentation of technological artistic production – Santa Maria, RS, Brazil, 2023.

Experimentation means testing the product of human awareness to apply it in the practical universe to which it is intended. Submitting the creation to experimentation is the opportunity to assess whether the product has quality technical- scientific content and whether it meets the target audience’s needs. Experimentation seeks to give legitimacy and credibility to what was created. It can be developed in two environments: institutional, in person and/or remotely, with the collaboration of expert judges and/or target audience, and practical context, aiming to reach the target audience (Chart 2).

### Applying the Created Product: Revolutionary Phase

In the product application phase, practice (application and technological assessment) can be understood as revolutionary. Becoming praxis, it demonstrates transforming social potential to people and contexts.

The technology initially forged in/by practice, returns to its origin and denotes meaning(s), whether in the organization of work, to mediate/facilitate relationships, strengthen (self)care, accelerate work processes, standardize practices, facilitate thinking diagnosis, strengthen educational processes, among others. Thus, it contributes to nurses’ managerial, care and educational praxis.

When returning to the practical scenario, the artistic production must be used by its target audience, in order to manifest its “practical potentials”: creative and reiterative (referring to the degree of creation) and reflective and spontaneous (referring to the degree of awareness – use). They vary according to each individual’s degree of awareness of the practical process, i.e., the way in which technology is used.

Creative praxis potential – creation is seen as an ideal object; proposes changes in reality through raising awareness; contributes to an autonomous and critical practice; reality changes as the object is used; allows facing new needs and situations; creation and context are intertwined, interacting and evolving.

Reiterative praxis potential – does not recognize artistic production as valid for its reality; the revolution does not take place, as the population prefers what already exists; creation does not produce change or transformation; it does not create possibilities for thinking and acting; reality remains the same; the public chooses to expand what is already created and being used.

Spontaneous praxis potential – it is not similar to reiterative praxis, as it manifests practical awareness; creation is used without manifesting reflections in man; its use is mechanical, it occurs involuntarily; the benefits of the object do not represent transformation in the practical process.

Reflective praxis potential – instigates the public to think in order to act; presents high levels of praxis awareness (theory and reflection on the practical process); reflection on practical activity encourages individual, collective and contextual change and transformation.

## DISCUSSION

### Understanding the Phenomenon of Interest: Pragmatic Phase

The interest in the interpretation of a given reality lies in the possibility of its transformation through the proposition of a technology. Experiencing the scenario becomes an intentional act aiming to understand its dynamics and identify the potential audience, and becomes the first step in the search for technological creation^(1,16)^. Reality is not susceptible to immediate acquisition and its reproduction requires specific knowledge and skills. The important thing is not what is seen, but what is seen with a method, because the researcher can see a lot and identify little and can only see the confirmation of their conceptions^(1)^.

For the ideal acquisition of reality, the pragmatic phase is inserted as a set of guiding elements for researchers to experience the practical process, assisting them in the design of research hypotheses, interpretation of reality, theorization of technological artistic production, for realization of knowledge.

In this phase, through pragmatic interpretation, researchers have an approximate idea of what needs to be created. However, it can be relative and merely guiding, since, while it is based on the hypotheses already outlined (derived from scientific evidence and/or observation of reality), they may undergo changes, given the frequency with which hypotheses and/or theories are put forward the evidence, sometimes being modified or refuted^(17,18)^. The insertion in the real scenario often symbolizes the experimentation of the new, asking the researchers to improve two mental operations: critical analysis and reflective synthesis (Figure 3).

Through critical analysis, a given reality, said to be complex due to its multidimensionality, should be reduced to simpler and measurable elements. In this way, it becomes possible to estimate and/or assimilate the whole from the information representativeness^(1,19)^. The pragmatic phase takes place, above all, through analytical and critical procedures. In the reflexive synthesis, data will be gathered considering their diversity, concreteness and/or abstraction, in order to be grouped into a coherent whole. When questioning reality^(20,21)^, researchers outline hypotheses, which must be testable and robust to propose a substantial scientific experiment.

Pragmatic interpretation suggests the creation of spaces for reflection and criticism with/about the context, using interpretive techniques^(20)^ such as hermeneutic circles, conversation circles, focus groups, among other strategies allowing interaction with the target audience and aiming to make them talk about subjects related to lived reality. Such subjects must emerge from the practical process^(1,7)^ through dialogues, attitudes and/or group dynamics, and must have meaning for the public, for the researcher and for the object of investigation. In order to better understand this practical process, researchers need to be part of it, have their insertion justified in the context of investigation, actively interacting.

Theorization permeates the pragmatic phase^(20–22)^, a complementary element in the construction of knowledge. Combining practice with theory^(1,16)^, it becomes essential to support thinking, reflecting and criticizing the practical process. Inserting the theory^(19)^ in the development of technologies allows the analysis of doubts regarding the result. Theorizing will be the path of coping between reality (global experiences and similar to what is being investigated) and idealization (product of human awareness capable of solving a given practical problem)^(1,16,19,20)^.

### From the Real to the Ideal: Artistic Productive Phase

The act of producing corresponds to the levels of consciousness established by men during the (inter)relationships with their universe, allowing the creation of various objects/tools useful for their daily needs. This process is only established in certain social conditions, called “production relations”. To produce, human beings use appropriate instruments and/or means, aiming to create, modify or transform something in light of a specific purpose. As a certain end materializes, it aims in a way a product derived from human awareness^(1,16)^. To subsidize artistic production, a guiding planning is proposed:

**Ideation**: the first production relationship allows inventors and the target audience to establish the necessary relationships to collectively think about the scope of what they want to create, i.e., what design will be adopted for artistic production. The prototype can be printed on paper, digital or mechanized, varying with the context’s needs. A study^(6)^ on the development of a product technology for the hospital setting used the brainstorming strategy with the nursing team to build the elements of its creation. Through meetings, the working groups validated the technology characteristics, later revised based on specific literature.

**Feasibility**: concurrently with ideation, inventors may face doubts and concerns about the feasibility of the proposal regarding its execution time, its scope, its content and the quality of the incorporated features/elements, its execution cost, among others. From the target audience’s feedback, questions may arise regarding creation design, its benefits, challenges for usability and cost of acquisition.

To make technology feasible, communication between inventors and context will make it possible to outline the structure/characteristic of what one wants to create, considering the audience’s knowledge and practices. Through theorization, a technical-scientific basis will be sought for artistic production as well as allowing inventors to carry out a cost-benefit analysis of the proposal.

**Partners**: after artistic production ideation and feasibility analysis, inventor(s)/researcher(s) must think about who their research partners will be. This step will have the participation of an interdisciplinary team composed of programmers, artists, writers, designers and other professionals with expertise in the areas corresponding to the creation of technology. The work must be carried out collectively, considering the target audience’s suggestions.

**Goals/deadlines**: From ideation, it is up to inventors to maintain the organization of their functions with a view to optimizing the work process, creating strategies to control activities, execution and delivery deadlines for each demand of the creative-artistic context.

**Resources**: in this step of production relations, there is the opportunity to plan which physical, material and financial resources will be involved in the creative process. After delimiting the scope of creation, inventors and partners must agree on the characteristics/elements for the prototype materialization. A study^(6)^ that built product technology for nursing care in the hospital context delimited the material resources needed for creation, considering legislation for the health area and guidelines of the Brazilian Association of Technical Standards (ABNT – *Associação Brasileira de Normas Técnicas*) and its Regulatory Standards (RS). The technology incorporated features such as a stainless steel structure, an electrical, mechanical and sensory system, and each raw material was linked after a careful market analysis to assess cost-benefit and feasibility.

The presented planning is interpreted according to the creativity potential^(23)^ to seek innovative solutions aimed at the real problems of a scenario, prioritizing creative solutions. Authors^(23)^ perceive the need for the act of creating to involve all human potential, as all people are creative and capable of contributing with relevant ideas. Thus, to produce in this participatory context is to place the different subjects (patients, family members, health professionals, managers, institutions, among others) at the center of the development of a solution and not just as users.

### The Practical Object Experimentation: Experimental Phase

Experimental scientific activity is evidently a form of praxis. It is an objective activity that generates a real product or result (technological tool). The experimental phase allows researchers to (re)assess the research hypotheses in the search for the ideal result (validated product). It is the opportunity to conduct the experiment already outlined into the practical field with a view to proving a theory or a certain aspect of it^(1,16)^.

The experimentation has a scientific, theoretical and systematic character, aiming to prove the hypotheses outlined by the research^(1)^. Under the context of praxis, it becomes the opportunity to submit the artistic production for assessment by judges in order to modify it according to the conditions in which a phenomenon takes place. In summary, the product resulting from the practical process is submitted to validity by the target audience and/or experts in the phenomenon.

The experimental phase represents submitting the creation by experts to validity in order to guarantee reliable indicators^(15)^. Submitting technological products becomes an expanding strategy to provide higher quality research, reduce methodological biases, ensure more accurate analyzes and achieve excellence in artistic production^(15)^. During experimentation, researchers must use instruments with reliability and validity to reduce potential subjective judgments on a given object.

### Changing Men and Their Social Context: Revolutionary Phase

Under the bias of philosophy, the revolutionary phase seeks a praxis solidified in practice. Such praxis aims to insert in the practical process a product (technology) of significant improvement for society, which seeks to change contexts. This praxis projects a futuristic character, i.e., it seeks to analyze the variables of application, usability and effectiveness of a given creation, considering the future of society in the face of artistic production^(1,16,22)^.

Revolutionary praxis is based on ethics and aspires to live well with/for others in an equitable way. The revolution of a practice lies in modifying social circumstances and the human being itself, seeking the transformation of man for them to become an agent of transformation of their context. Individuals are conditioned by the social situation in which they find themselves. Accepting these premises is indispensable for revolutionary practice, born of the contradiction between the productive forces and the relations of production^(1,16)^.

In the face of technological assessment, to achieve the revolution of reality, more than philosophy is needed, philosophy needs to be realized in action for change, transformation, evolution of theory and practice. This movement happens only through the mediation proposed by praxis^(1,22)^. When philosophy abandons its purely theoretical character and becomes practical, it is capable of becoming a transforming force in reality.

Praxis takes place when criticism enters the awareness of men and becomes effectively a force. Therefore, society must mediate this process and this requires a critical understanding of reality and the conversion of criticism into action and into revolutionary praxis. It must be radical in the sense of seeking in men the central object of its analysis, corresponding to radical needs, and it must start from them and for them as a mediating link between philosophy and reality^(1,16,22)^.

In the revolutionary phase, artistic production reaches its praxis potential as it acquires the ideal theoretical and practical sense, being able to instigate practical awareness and/or praxis in the search for transformation. This change will only occur if theory and practice are (co)related^(1,16,22)^. Thus, during the practical revolution (application of artistic production), the theoretical revolution must be present, becoming a tool for validating the hypotheses and theories contained in the creation.

The model in question has as a limitation that it has not yet been tested, through its use, with a view to evaluating its flexibility and usability in different environments and health conditions.

PMTD will contribute to the advancement of scientific knowledge as well as of nursing as a science, giving researchers the opportunity to operationalize its phases, constituted with evident onto-epistemological bases^(4)^. The model, through its dialectical and systematic structure, will allow the conception of new technologies in order to make them meaningful, applicable and usable by the practical context to which they are intended, in order to reach the expected practical revolution.

## CONCLUSIONS

Men have in their essence “being a creator”, even though they do not live in a constant state of creation. Nursing has created (new technologies) to solve emerging needs in their daily lives. Therefore, praxis comprises consciously oriented practice, which, combined with theory, allows them to transcend their thinking and acting to analyze, interpret and intervene in the practical process. Bearing this in mind, PMTD demonstrates relevance and potential in helping nursing researchers and related areas in the development of products and/or technological processes to redefine their practical activity.

PMTD, through its theoretical and philosophical basis of praxis, allows researchers to understand realities, identify problems and propose solutions from a participatory perspective. Its methodological structure in four phases brings operational and epistemological content for artistic production construction, validity and assessment for the practical context.

The model in question becomes an important tool for solving emerging problems in nursing and health professionals’ practice, and can be applied in different professional settings.
